# International Health Electives: defining learning outcomes for a unique experience

**DOI:** 10.1186/s12909-023-04124-4

**Published:** 2023-03-15

**Authors:** M. A. C. Versluis, N. C. Jöbsis, A. D. C. Jaarsma, R. Tuinsma, R. Duvivier

**Affiliations:** 1 Department of Obstetrics and Gynaecology, University Medical Center Groningen, PO Box 30001, 9700RB Groningen, the Netherlands; 2 Rijksuniversiteit Groningen, Faculteit der Medische Wetenschappen, Groningen, the Netherlands; 3 Center for Educational Development and Research in Health Sciences (CEDAR), Lifelong learning, Education and Assessment Research Network (LEARN), University of Groningen, University Medical Center Groningen, Groningen, the Netherlands; 4 Faculty of Medical Sciences, University Medical Center Groningen, Groningen, the Netherlands; 5 Faculty of Veterinary Medicine, Utrecht University, Utrecht, the Netherlands; 6 International Office, Wenckebach Institute for Education and Training, University Medical Center Groningen, Groningen, the Netherlands; 7 Parnassia Psychiatric Institute, The Hague, the Netherlands

**Keywords:** Global Health Education, International Health Elective, Low resource setting, Competency development, Learning outcomes

## Abstract

**Background:**

An International Health Elective (IHE) can be a unique learning experience for students. However, it has proven difficult to clearly define learning outcomes that capture the complexity of an IHE and are aligned with future professional performance. This study aimed to further define learning outcomes for IHEs in low- to middle-income countries (LMIC) from a student perspective.

**Methods:**

We conducted a deductive analysis of pre-departure and post-elective reflective reports of fifth-year medical students who participated in an IHE as part of their program. This provided possible learning objectives that were further explored in semi-structured individual interviews with medical students who had recently returned from an IHE.

**Results:**

We analyzed 33 reports of students participating in an IHE from 2017–2019 and held 19 interviews. Thematic analysis revealed 9 themes: developing intercultural competence, developing appreciation for differences in health care delivery systems, understanding international health, understanding the global burden of disease, developing a career perspective, developing clinical skills in resource low settings, becoming cost conscious, developing social responsibility and self-actualization.

**Conclusions:**

We identified 9 learning outcomes that are directly and indirectly related to clinical practice. They add to the on-going discourse on the benefits of IHEs. These outcomes can be further developed by investigating the perspectives of home and host supervisors and educationalists, while taking the local context into account. Follow-up studies can evaluate to what extend these outcomes are achieve during an IHE.

**Supplementary Information:**

The online version contains supplementary material available at 10.1186/s12909-023-04124-4.

## Background

We live in a rapidly changing world where mobility is increasing and countries are ever more connected and interdependent. The SARS-CoV-2 pandemic not only illustrates the contagiousness of a virus, but also the modulating effects of mobility, behavior and culture. It demonstrates that being connected and interdependent requires an international approach to healthcare and health professions education. The incorporation of global health into (medical) education is of growing importance to resolve future challenges in healthcare that extend beyond national borders [1]. As such, Global Health Education (GHE) has been receiving increasing attention in the scientific literature, with numerous studies describing the themes, competencies and learning objectives relevant to GHE [2–5]. Several institutes such as the Global Health Education Consortium and the Consortium for Universities for Global Health address GHE [6, 7]. They underline the need for further development of GHE on the basis of future healthcare needs and educational needs of students. However, there is limited scientific evidence or grey literature describing which learning outcomes are most relevant to GHE. This paucity in our understanding hinders appropriate allocation of resources, as there are inherent pitfalls in GHE, particularly around short-term (elective) placements in low-resource settings. When done irresponsibly, there are significant ethical issues and potential risks for host institution, host population and participating students [8–10]. This study aims to make a contribution to our present understanding by defining and specifying learning outcomes in the setting of an international health elective for undergraduate medical students.

A well-known example of GHE is an International Health Elective (IHE) where students travel abroad for part of their training. An increasing number of students participate in IHE programs, in most cases traveling from a High Income Country (HIC) to a Middle- or Low Income Country (LMIC) [1–5, 11]. Research on the impact of an IHE revealed several benefits [12–18]. Some of these benefits are directly related to clinical practice. For example, in a setting where diagnostic options such as x-rays or laboratory tests are limited, clinicians and students are forced to rely on findings upon physical exam which may benefit the development of examination skills. Other benefits are not directly linked to clinical skills or practice. For example, anecdotally, upon their return from IHE, students mentioned that it had “broadened their horizon” and that they had “gained an understanding of culturally different perspectives on disease”. Although these benefits are not directly linked to clinical practice, they can be very valuable for professional practice as a healthcare worker. In a study describing the benefits of on the outcomes of international mobility programs for higher and postgraduate education in general, Roy et al. [12] discriminated between benefits that are directly or indirectly related to professional practice and classified three areas of outcomes: cultural, personal and employment/career outcomes.

Specific to students in health professions education, a broad range of benefits of an IHE has been suggested [13–23], including gaining knowledge about tropical diseases and healthcare systems, learning to deal with limited resources and developing cultural competences. Battat et al. [18] performed an extensive literature review and provided an overview of the benefits of an IHE by describing 14 competencies [18]. Although this review provided an important step towards describing possible learning objectives of IHEs, there were some important limitations. For example, the included studies used a variety of competency-based approaches to GHE, often without a clear definition of what these competencies entailed. Studies in other health professions education such as nursing and pharmacy describe a similar challenge in defining learning outcomes for an international health elective [20–22, 24]. In another study, Nordhues et al. [16] analyzed reports of students who had finished an IHE and related the competencies they found to the core competencies described in the Accreditation Council for Graduate Medical Education (ACGME) framework. They identified 24 themes that fit within this framework. Although this framework is well defined, it is based on themes that are relevant to and developed in a high resource setting. Consequently, important aspects relevant to the specific setting of an IHE can easily be missed, which might make this framework less applicable to IHEs.

The current understanding of IHE learning outcomes limits their incorporation into GHE [2, 3, 18]. The underlying studies by Roy et al. [12] and Battat et al. [18] yielded a large variety of outcomes with no clear definitions of threshold concepts. As a result, it is difficult for students and educators to define learning objectives for a specific IHE and explain how the experience can contribute to professional practice. A further understanding of possible learning outcomes, both directly and indirectly linked to clinical practice, can facilitate incorporation of IHEs into GHE and help clarify their link to professional performance [1–3].

This study aims to further clarify the possible learning outcomes of an IHE as perceived by students. We adopted the student perspective as students are participants in IHEs and, as such, have a lived experience of being on placements. Since our aim was to explore and elucidate learning outcomes for IHEs, we performed a qualitative study with individual semi-structured interviews based on written reflective reports and existing literature [12, 18].

## Methods

### Design

We performed a qualitative study using pre- and post-departure reflective reports as well as semi-structured interviews with medical students detailing their experiences with an international health elective in LMIC. The initial findings of the reflective reports informed the semi-structured interviews. A study protocol was formulated, guided by current perspectives on methodology and reporting [25]. We used directed content analysis to identify relevant themes [26–28].

### Setting

The undergraduate medical curriculum of the University Medical Center Groningen (UMCG) takes six years and comprises a three-year Bachelor’s and a three-year Master’s program. The programs are problem-based and patient and student centered. The Master’s program includes 2,5 years of clinical rotations and 0,5 year of academic research and writing a master thesis. Fifth and sixth-year medical students have the opportunity to do an IHE abroad for a period of 8 to 12 weeks. Partner organizations receiving these students are located all over the world, mainly in LMIC settings. During an 8-week elective, students either focus on public health or on tropical medicine (including infectious diseases, obstetrics/gynecology and surgery). They can also opt for a 12-week elective combining both topics. All participating students follow an obligatory preparatory course addressing medical topics such as tropical diseases, public health, reproductive health and pediatrics as well as topics related to cultural awareness, logistics (obtaining funds, visa etc.) and safety issues (insurance, prevention of and response to emergencies). The course consists of 6 lectures of each 2 h and is finalized by an online exam.

### Participants

Students who participated in an IHE between 2017 and 2019 were asked for permission to use their pre-departure and post-elective reports. This cohort was chosen because a new format for pre- and post-departure reflection was implemented in 2017. In their reports, students were asked to reflect upon their expectations before and experiences after an IHE. Students were prompted to reflect on items such motivation, goals and expectation before departure using a form with open questions.. After the elective, students were prompted to reflect on the program, content and recommendations, travelling, safety, finance using a form with open questions. A brief survey investigated their experience, achievement of learning objectives and supervision using a Likert scale. Forms for pre- and post-departure reflective reports are added as supplementary material. Students received an information letter explaining the purpose of the study and stating that all data would be handled confidentially, that anonymity was guaranteed and that they were free to withdraw at any time. All participating students provided written informed consent. Subsequently, the primary researchers received anonymized pre- and post-departure reflective reports from the international office facilitating the electives. Students who had returned from their IHE since the beginning of 2019 were asked to participate in an individual, structured interview. The same informed consent procedure applied to these students.

### Data collection and analysis

Analysis of the reflective reports informed the semi-structured interviews. Both the reflective reports and subsequent semi-structured interviews were analyzed using a deductive approach [26, 28]. We started with analyzing students’ reflective reports of the 12-week combined elective, as it was likely that the outcomes of an IHE would be more clear for students who spent a longer period of time abroad [29, 30]. Subsequently, we analyzed the reports on the 8-week tropical medicine or public health internship. Pre- and post- departure reports were analyzed as whole. Relevant topics students discussed in their reflective reports and their statements related to possible learning outcomes were categorized using directed content analysis [26, 28]. First, the studies by Battat et al. [18] and Roy et al. [12] were used to define initial coding categories. Because we wanted to include learning outcomes that could be linked both directly and indirectly to clinical practice, we used the three outcome domains as described by Roy et al. [12] as a starting point for our framework. Building on these domains, we included the 14 GH competencies that are more specific to health professions education to refine our framework [18]. Although other studies in learning outcomes of an IHE have been published after the study by Battat et al., this study provides an extensive analysis of literature from several perspectives. Some competencies could be related to more than one domain and would be further explored in the interviews. We used the framework as the basis for developing an initial coding scheme. Next, reports were screened and descriptions of possible learning outcomes for an IHE were highlighted by the primary researchers (NJ and MV) [28]. Highlighted information was categorized using the initial coding scheme. Coded descriptions that could not be categorized into any of the existing categories were used to establish new categories. Primary researchers discussed each possible learning outcome. When there was disagreement, the outcomes was discussed in the research group to reach consensus. In an iterative process, coding and categorization were discussed during regular research team meetings and adapted where necessary. The process continued until all possible learning outcomes had been categorized.. After that, the results of this analysis –i.e. a list/number of possible learning outcomes– informed the semi-structured interviews with students who had returned from their IHE.

The interviews served two purposes. First, to provide a more in depth analysis of students’ statements to further refine the formulation of definitions of learning outcomes. Second, to allow students to consider their motives for choosing an elective in a LMIC as well as to deepen their reflections on their experiences. We aimed for a sample size of 15 respondents since we used the interviews as secondary data to evaluate and apply the insights gained from the reflective reports [31]. All interviews were tape-recorded and transcribed verbatim (by NJ). Member checking was used to validate the researchers’ interpretation of the interviews and give the student the opportunity to make additional remarks [27, 32]. Coding and categorization into learning outcomes were discussed during frequent research team meetings, again in an iterative process. This process allowed to further specify and narrow down the possible learning outcomes following from the analysis of the reports. Atlas.ti software for Windows (version 8.4.16) was used to facilitate data analysis.

### Ethical considerations

Ethical approval was obtained from the Ethical Review Board of the Netherlands Association of Medical Education (NVMO, A 2019.4.5). To protect student privacy all reflective reports were distributed anonymously to the researchers. All participants gave their informed consent and could withdraw from the study at any time.

### Reflexivity

The research team consisted of a medical student (NJ), a global health expert (MV), a social science graduate and employee at the international office (RT) as well as educational researchers (AJ and RD). MV served as a daily supervisor for NJ. Regular meetings with the complete research group were organized to discuss the research process and analysis of data.

## Results

### Analysis reflective reports

**Fig. 1:**
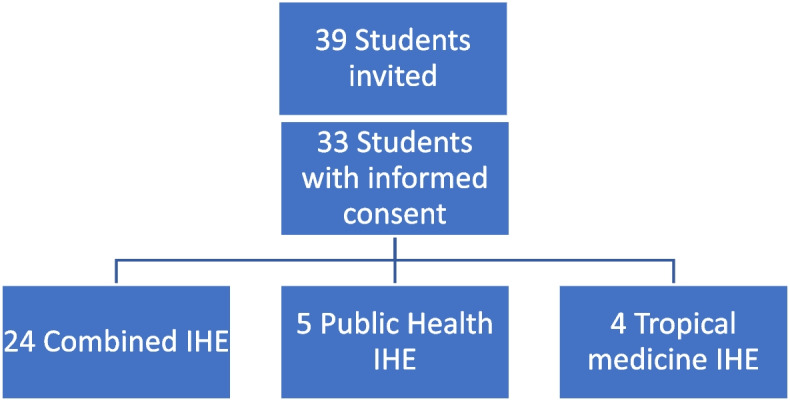
Inclusion of reflective reports

Contact information was available for 39 students, who were all invited to share their reflective reports (Fig. 1). A total of 33 students gave permission to analyze their reports (85%). Reports on the tropical medicine internship, public health internship and the combination internship were all taken in to account. The period in which the IHEs took place was from July 2017 to February 2019. Internships took place in the following countries: Ghana, Indonesia, Malawi, Mozambique, Nepal, Nicaragua, Uganda, Surinam, Tanzania, Zambia and South Africa.

Deductive analysis of provided 20 categories of possible learning outcomes relevant to an IHE. Table 1 summarizes these categories, including illustrative quotes taken from the reflective reports. 6 related to the cultural development, 6 to the employability/career, and 9 to the personal development domain. The possible learning outcomes covered most, but not all 14 global health (GH) competencies as described earlier [18]. Some learning outcomes related to more than one domain, which we further explored in the interviews. Experiences that related to the global health competencies and were mentioned by most students were: an understanding of travel medicine, skills to better interface with different populations, cultures and healthcare systems, appreciate contrasts between healthcare delivery systems and expectations and understand healthcare disparities between countries (Table 1). Four GH competencies were not mentioned in the reports: humanism cost of global environmental change, evolving global governance issues and scientific and societal consequences of global change. Seven possible learning outcomes had not previously been described and were included as new learning outcomes: training of (new) practical skills, career perspective, (learning) to set personal boundaries, gaining confidence in own (medical) actions, creativity in problem solving, reflecting on own behavior (at home and/or during elective), developing cultural awareness. These competencies had been mentioned as both learning outcomes prior to the elective and more developed competencies afterwards.

**Table 1:** 20 possible learning outcomes relevant to an IHE described in pre-post IHE reports. Ordered according to related domains (Roy et al. [12]) and GH competencies (Battat et al. [18])

**Competency domain (according to Roy et al.)**	**GH competency (According to Battat et al.)**	**Illustrative quote from reflective reports**
**Cultural development**	Scientific and societal consequences of global change	‘’Nicaragua is still developing a lot and I am curious [to find out] how this affects the health care system, what strengths and weaknesses are, and how people are trying to improve it as well as trying to see it from the perspective of the local population.’’
	Appreciate contrasts in healthcare delivery systems and expectations	“Sometimes I felt unprepared and I was very surprised about the course of events: the limited resources and treatments, the dismissal of mental illnesses, belief in herbal medicine … but you cannot prepare for this and you learn to deal with it during the experience you gain”
	Understand healthcare disparities between countries	‘’It added a lot to my personal development. I never worked in a developing country. It gave me a good insight into how to cope with less materials and medicines. The healthcare system in the Netherlands is well developed, and we can be proud about it. It was sometimes hard to see patients who couldn’t afford any treatment and there are still women dying of bleeding after pregnancy. This was shocking to see sometimes, but I would never have such an experience in the Netherlands.’’
	Primary healthcare within diverse cultural settings	‘’Learn from their culture and the way they function as a nurse or doctor. I also want to learn to handle cultural or religious differences in habits, opinions and interactions, both in a general way and in a medical setting.’’
	Skills to better interface with different populations, cultures and healthcare systems	‘’I learned how poverty and traditional medicine influence access to regular health care; the importance of proper health education on the decision-making of patients; a different culture with a different mindset [sic] about health and illness; dealing with death, as relatively many patients died of their illness.”
**Employability and career**	An understanding of immigrant health	“We also went to the nearby refugee camp a few times, where we helped out at the clinic and saw our own patients.’’
	An understanding of travel medicine	“Learned a lot about tropical diseases such as malaria, TB and HIV. For example, I experienced myself how patients with these diseases present themselves. I learned about the treatment, but also about possible additional complications and comorbidities [sic]. I would never have been able to learn this in the Netherlands, given that it does not occur very often. Even if I start working in the Netherlands, this is very relevant for me as a future doctor’’
	Cost consciousness; using physical diagnosis without high technological support	‘’The importance of the patient’s economic status and trying to reduce expenses showed me a new perspective, which I will always keep in the back of my mind. Their resourcefulness in some cases to reduce costs and help even the poorest patients is astonishing.’’
	Taking adequate patient histories and physical examination in resource poor settings	‘’My physical examination and clinical reasoning skills improved, both because it became one of the most important tools to rely on and because of the far advanced diseases I have seen.’’
	Training of (new) practical skills^a^	‘’For me the one thing that stands out is that I was able to see about twenty vaginal deliveries, plus another twenty caesarean sections. I would have never been able to see this in any Dutch hospital as a trainee.’’
	Career perspective^a^	‘’Prior to this internship, I had no ambitions to work abroad. This internship taught me that foreign healthcare has become more accessible to me. After this internship, the plan arose to go to Surinam for six months after completing my studies to gain work experience.’’
**Personal development**	Skills to better interface with different populations, cultures and healthcare systems	“During the preparation of our recommendations, we had to take into account the interests of all different parties….the financial and ethical aspects of this project also deserved the necessary attention. In addition, we had to take into account the local culture and vision of the local population in order to determine where the profit could be made.’’
	An understanding of the burden of global disease	“It has not changed my plans for the future, but I think it complements being a doctor and I now have a better understanding of why, for example, HIV is still such a big problem and that it is really not that easy to deal with.’’
	Develop a sense of social responsibility	‘’I know we cannot solve all the problems there, but I think we can help the community in Uganda with our knowledge.’’
	Evolving global governance issues	‘’A lot of things were not what they seemed or brought in a way to favor [sic] one opinion over the other. This made me realize very well that things are not always what they seem and that some people high up in the governments might be really bad.’’
	(Learning) to set personal boundaries^a^	‘’Intern doctors in Africa have a lot more responsibilities in the hospital compared to Dutch trainees, it is very important as a Dutch medical student to show your boundaries in treating the patients.’’
	Creativity in problem solving^a^	‘’It gives you great experience in improvising, but in a safe, medical way. A lot of medication here is not available so you have to really think what the next step is instead of just reading what treatment is supposed to be given and give it. The same goes for equipment, e.g. how to stabilize complicated fractures, how to keep a leg up in the air if the bed cannot be moved upwards, how to clean and dress wounds with only the normal gausses we have here.’’
	Reflection on own behavior (at home and/or during elective)^a^	“It put my feet back on the ground and made me realize that living the way we live is not the standard there, but how happy and cheerful people are without everything we have … I've learned not to just take things for granted.’’
	Gain confidence in own (medical) actions^a^	‘’I really learned to interpret nonverbal communication and focus on the way the patient was looking: ill-looking, fair, stable, pallor etc. thereby I learned to trust more on clinical judgment and use less advanced diagnostics, simply because it was not always possible there.’’
	Growth in cultural awareness^a^	‘’The internship also gave me a different perspective of the world. Hard working is important in the Dutch culture, but in the African culture it is more about communication/community and family. There is more in the world than working. This was an eye-opener for me.’’

^a^Description of new competency. The quotes support and illustrate the accompanying competency and shows the kind of statements given by students in the reflective reports

### Analysis of individual interviews

To further investigate and refine the 20 possible learning outcomes found in the analysis of student reports, 46 students were invited to participate in the semi-structured interviews. 39 students responded, of whom 21 were willing to participate (46%). Because of distance, 2 students were unable to meet with the interviewer and were excluded. A total of 19 interviews were conducted. The average age of students during their IHE was 24.6 years and three male and sixteen female students were interviewed. Electives took place in Ghana, Malawi, Nepal, Rwanda, Uganda, Sierra Leone, Surinam and South Africa. Within this group, 15 students went on a tropical medicine IHE, 1 on a public health IHE and 3 on a combined IHE.

Using the interviews, the 20 possible learning outcomes were further refined, resulting in 9 clearly formulated learning outcomes which will be described below. The outcomes are ordered by domain: cultural development (C); employability/career (M); and personal development (P). Some of the learning outcomes relate to more than one domain.

#### Developing intercultural competence (C,P)

Students get acquainted with a new culture and learn how to work within this different cultural setting as well as how to approach people/patients in an appropriate manner (also called intercultural communication). Students learn to solve a problem, taking into account all parties involved and their sometimes different interests, while respecting local norms and values. Furthermore, students learn to open up to a new culture and/or different professional habits and, in the meantime, they learn how to work with these differences in mind. Students experience a different way of life and a different culture in the host nation and institution. Conversely, the students reflected that they had developed a new, different view of their own culture, behavior and (work) attitudes in Dutch society.

#### Developing appreciation for differences in healthcare delivery systems (C,M)

Students get to know the organization of the host nations’ healthcare system and gain an understanding of differences in healthcare systems. They learn to appreciate the influence of culture, religion, health literacy of the population and economics on both a healthcare system and the way patients and their environment cope with illness. They learn to work within a health care system with a different culture, manners and professional habits.

#### Understanding international health (M)

Students are introduced to (infectious) diseases which are rare in the sending country but common in the host country and learn why treatment of these diseases has proven to be so difficult. Additionally, they are confronted with diseases that are more familiar to them but present in a much more advanced stage. They gain knowledge on how to diagnose and treat these diseases by focusing on differences in diagnostics and treatments.

#### Understanding the global burden of disease (M)

Students gain insight in common diseases of the region and differences in prevalence of diseases between the host and the sending country. They learn to put the treatment choices into perspective and appreciate differences in treatment driven by a knowledge gap and/or low resources. Students learn about prevention programs and the impact of illness on the patients and their environment.

#### Developing a career perspective (M)

Students’ experiences abroad either confirm or discourage their aspirations for working abroad. Some students become more certain of their choice of medical specialty or their wish to be involved in healthcare projects in low income countries.

#### Developing clinical skills (M,P)

Because of limited access to diagnostic tests, students need to further develop their clinical skills needed for history taking and physical examination. Students feel that recognition of symptoms of disease and clinical reasoning are more profoundly trained. They learn to cope with different circumstances and to come up with practical solutions, for example, they train practical skills such as performing physical examination or assisting in the operating theatre. In addition, students work on professional communication skills as well as communicating in a different language inside and outside of the hospital and/or learn to communicate through an interpreter or using nonverbal communication skills.

#### Becoming cost conscious (M,P)

Students experience greater awareness of the costs of healthcare and learn to be more pragmatic because of a limited amount of resources. Students develop greater appreciation for the prevalence of health insurance and the influence of the economic welfare of a country on its healthcare system. Students gain more appreciation for the sending country’s healthcare system and all its possibilities. However, they also develop a more critical view on the consumption of diagnostic means and/or medications by medical doctors in the sending country. Students learnt to cope with differences in availability of treatment options and the sometimes unfortunate effects.

#### Developing social responsibility (P)

Students develop increased awareness of others around them and gain drive to improve a situation without self-promotion. It reminds them of their motivations to become a medical doctor. Furthermore, they understand that developmental aid only has a limited effect when it is for a short period of time and question how much impact their presence truly has on the host country/hospital. They become able to weigh the ethical dilemmas associated with their stay abroad such as the benefits of their presence for the local population/the hospital versus the local investment in their training.

#### Self-actualization (P)

As a result of the (large) differences between the circumstances in their host and sending country, students experience how to handle working outside of their comfort zone. This can increase their self-confidence regarding their own *action*s. At the same time, students learn to recognize and guard their own boundaries and how to indicate that they do not feel competent enough to perform a certain task. Because of interactions with people within and outside of the hospital, students learn more about their own behavior and attitudes as they get the opportunity to reflect and talk about it with the local population. Differences in cultural background, religion, attitudes and beliefs of the host country as well as political conflicts or economic status play an essential role in this new understanding.

Importantly, the participants often mentioned the importance of the local context and how it facilitated their development towards a specific learning outcome. For example, in countries where students were more familiar with the language, such as Surinam or South Africa, they were more able to communicate and work independently. This facilitated development of medicine-related learning objectives such as developing practical skills and gaining an understanding of international health. In countries such as Nepal or Uganda, where students suffer more from a language barrier, students felt they learned more in terms of personal and cultural development because they were forced to focus more on non-verbal communication and context to understand what was happening. Furthermore, students participating in a tropical medicine IHE or a combination internship IHE more often described development towards medicine-related learning outcomes, whereas the student who participated in the public health internship felt that cultural competencies had been further developed during the elective. Students also stressed the importance of learning objectives within the domain of personal development, which are unique to an IHE. Important aspects within this development domain were gaining more self-confidence, learning to put things into perspective, developing greater appreciation for the conditions in the sending country and learning to care more for the people around them and gaining awareness of the differences in lifestyle and living situations around the world.

In the interviews, students also mentioned non-educational motivations for choosing an IHE at a specific location, for example, the desire to experience life abroad for an extended period of time (more than a vacation), having lived abroad before or having enjoyed a previous visit to the country/a LMIC and having the desire to return.

## Discussion

In this study, we aimed to refine and extend the learning outcomes that are relevant to an IHE and improve definition of threshold concepts that will facilitate incorporation into GHE. We formulated 9 learning outcomes: *developing intercultural competence, developing appreciation for differences in health care delivery systems, understanding international health, understanding the global burden of disease, developing a career perspective, developing clinical skills, becoming cost conscious, developing social responsibility and self-actualization*. These learning outcomes deepened our understanding of how IHEs can relate to clinical practice, either directly or indirectly. Our results are consistent with those of other studies but also complement them in breadth and depth, offering insights into how these learning objectives can contribute to professional performance [12, 16, 18, 20, 22–24].

Although the 9 learning outcomes described here share similarities with the GH competencies described by Battat et al. [18], some differences stand out. For example, GH competencies such as *humanism* or *cost of global environmental change* were not identified in our study. A possible explanation is that our students may not be aware of such a learning experience taking place, or may not have been able to verbalize experiences that fall within these competencies. For example, they may not have realized that a learning experience such as supporting a patient in dire distress is related to humanism. Alternatively, it could be that learning outcomes described in previous studies did not apply to each student’s specific experience. In addition, the learning outcomes we formulated are not mutually exclusive, but closely related. For example, *developing social responsibility* can also be considered a form of *self-actualization*. This interrelatedness underlines the complexity of an IHE and is shared with other health as well as non-health profession [20–24]. For example, *developing intercultural competence* relates to the cultural and the personal area as described by Roy et al. [12]. Similarly, Schellhase et al. describe how differences in culture and patient care, trigger reflection and personal values and beliefs resulting in progress in cultural sensitivity [24]. This illustrates the interrelatedness of learning outcomes. In addition, it illustrates that some learning outcomes are not limited to a specific health profession. Rather, learning outcomes that address more general competencies such as cultural competency apply to a broader audience, within and outside health professions education. With increasingly multicultural societies this competency is of growing importance. A similar point can be made for competencies such as *understanding international health, understanding the global burden of disease, becoming cost conscious and developing social responsibility* that are of growing importance because of societal developments such as globalization and rising health expenditures [12].

Students also emphasized the importance of local context in obtaining different learning outcomes. Peluso et al. stated that describing competencies and learning outcomes is essential for developing and implementing a GHE, but they acknowledged that these competencies can hardly cover all aspects of an IHE [3]. They argued that an IHE has the potential to facilitate transformative learning by encountering learning experiences, but stressed the need for an experience to be informed by the local context. We sought to describe the learning outcomes clear enough to assist learning and broad and flexible enough to be adaptable to local contexts and individual students’ educational needs. Exploring the relationship between the learning outcomes and local context was beyond the aim for this study but is an important subject for future studies.

In order to align learning objectives with the local context, we need to understand the learning opportunities at receiving institutions [3, 4]. Roebbelen et al. [8] investigated reflections of local supervisors on the impact of IHE’s and briefly addressed some perceived learning outcomes for students. They described students’ distress when they were confronted with health inequities and a different socio-economic context, culture and healthcare system. This is in line with the learning outcomes as perceived by the participants of our study. The authors also emphasized the importance of a thorough predeparture training to prepare students for an IHE. Teachers and educationalists from institutions sending students abroad, especially those with a background in GH, can play a key role in developing such a training since they have medical/educational expertise and have already experienced the inequities students face during an IHE. Our understanding of learning opportunities from the perspective of teachers, both sending and receiving teachers, is still limited and needs to be further explored.

### Strengths and limitations

The deductive, iterative analysis of reflective reports and consequent semi structured interviews allowed the researcher to obtain an in depth understanding of the possible learning objectives of an IHE. The retrospective nature of our study, focusing on student perceptions and experiences, may have introduced a form of recall bias which may have affected the completeness of the data provided. This was mitigated by using a variety of sources, including the pre-departure and post-elective reflective reports and individual interviews. Due to the nature of our recruitment process, we may have inadvertently allowed for selection bias, since we cannot verify the experiences of the non-participants. Our sample size, iterative approach to data analysis and the fact that recruitment continues until we reached data saturation may alleviate these concerns. Finally, this study included students from a specific context (University Medical Center Groningen, the Netherlands) visiting a diversity of locations. Although analysis was continued until data saturation was reached, findings in a different population of students, visiting different locations may be different.

### Further research

An IHE offers a unique opportunity for students to develop clinical skills and competencies that are not directly linked to clinical practice but relevant to later professional practice. To optimize this learning opportunity, we need learning outcomes that are specific enough to be implemented in GHE, but also broad and flexible enough to be adaptable to local context and individual students’ educational needs. This study adds to the current understanding of IHE learning outcomes by describing all possible learning outcomes as perceived by students. Students are in the unique position to comment on their experiences as learners. However, future research also needs to focus on the perspectives of other stakeholders, such as local supervisors in the receiving and educationalists in the sending country. Further research should also explore the IHE learning outcomes and the extent to which students have achieved them, in prospective implementation studies. A next step is to explore other stakeholders’ perspectives to further develop the learning outcomes and elucidate in which context which learning outcomes are important for which students. Follow-up studies can evaluate to what extend these outcomes are achieve during an IHE. Finally, in line with the GH objective of achieving health equity and sustainability, it is important to consolidate international educational partnerships addressing reciprocity in development or redesign of IHE programs [3, 4]. Future research should investigate the conditions and requirements to secure sustainable, reciprocal partnerships.

## Conclusion

We described 9 learning outcomes for IHEs that can prospectively be implemented to guide GHE. Our findings stress the importance of taking the local context of an IHE into account. Since there is a broad variety in individual students’ backgrounds and local contexts of IHEs, not all 9 learning outcomes may be applicable to each elective. Alternatively, there may be other learning outcomes that our participants may have overlooked. Further research can help elucidate which learning outcome is relevant to which context.

## Supplementary Information

**Additional file 1.**

## Data Availability

The datasets used and/or analyzed during the current study are available from the corresponding author on reasonable request.

## Abbreviations

MV: Marco Versluis
NJ: Noor Jöbsis
AJ: Debbie Jaarsma
RT: Renzo Tuinsma
RD: Robbert Duvivier
IHE: International Health Elective
GHE: Global Health Education
LMIC: Low/Middle Income Country
HIC: High Income Country
ACGME: Accreditation Council for Graduate Medical Education
UMCG: University Medical Center Groningen
NVMO: Netherlands Association of Medical Education
GH: Global Health
