# Supplementary material for: International Health Electives: defining learning outcomes for a unique experience
Source: BMC Med Educ. 2023 Mar 15;23:157. doi: 10.1186/s12909-023-04124-4 (PMC10015142; doi:10.1186/s12909-023-04124-4)
Supplement: Supplementary file 1 — Additional file 1. [file 12909_2023_4124_MOESM1_ESM.zip › umcg-application.pdf]

2022 - 2023

UMCG Grant Application

Introduction & general instructions

Dear student,

This application form for a UMCG Grant consists of 2 parts:

- Part A is for *formal* purposes, in order to be able to transfer the advance part (70%) of the grant. This part is only for internal use by the international office and the financial department officer, and not to be shared with any other student or staff member.
- Part B is about *content, learning goals and expectations*, as point of reference for the report afterwards. So please keep a copy for yourself for this purpose!

The application needs to be filled in *personally*, also in case of joint or group work. Part 2 of the application form might be partly identical for group work, not for individual internships at the same location.

Reporting afterwards is mandatory, within 30 days after the end date of the mobility activity; failing to comply may also lead to reclaim the advance payment (70%).

If you have any questions (or feedback) please address fwm.naarhetbuitenland@umcg.nl

**Along with this document (part A+B!) add the following documents - appendices**
• **Study/ Internship Abroad Form**
• **Written invitation/acceptance proof confirming *period* and *supervision* by the receiving institution**
• **Approval for specific study item / content (from internal supervisor / examiner / teacher / coordinator / committee)[1]**
• **For clinical activities/internships: proof of sufficient language knowledge/skills for specific location[2]**
• **For clinical activities/internships in low income countries: copy of certificate Tropical Medicine preparatory course ('Tropencursus')**
• **Indemnity declaration**

[1] From UMCG or Affiliated Hospital Commission OMW, SAS or examiner THK or contactperson Internationalisation BW

[2] Depending on destination, please see for more information: https://student.portal.rug.nl/infonet/studenten/umcg/geneeskunde/studieofstageinhetbuitenland/watmoetikregelen/regelenvooraf/taaleisen

UMCG Grant Application Form – Part A

Deadline to hand in this form: at the latest 6 weeks before start date mobility
Please fill in electronically and send as one Word-file, together with part B and Annexes (see above), to fmw.naarhetbuitenland@umcg.nl, in the subject line *'UMCG Grant Application'*

**1. Personal & contact details**	
First & Family name	
Student number	
IBAN Bank account number	
BIC/SWIFT code	
Name of the bank	
City where your bank is located	
**2. Health insurance**	
Insurance company	
Insurance number	
**3. Grants previously awarded**	
Received a grant before?	☐ Erasmus grant<br>☐ Marco Polo grant<br>☐ UMCG grant<br>☐ Neither of these

**9. Signature**	
Name	
Place and date	
Signature[3]	

[3] Please paste as image/picture

Privacy Statement
Under no circumstances will your personal data be disclosed to any other individuals or companies outside the University of Groningen.

UMCG Grant Application - Part B

Save a copy for yourself, as point of reference for the report afterwards

**Motivation, goals and expectation [4]**
**Why did you choose to do this study activity at *this* location/hospital/ institution, in *this* country? E.g. what are the *specific* learning aspects for you *for this destination*?**
**Your personal (or collective) learning goals may be of first priority, but please also consider the perspective of the host. In what way do you expect to be of benefit to the receiving institution? What do you intend to bring/contribute in return?**
**Procedure and admission**
**Did you arrange for this institution yourself, or did you get advice and/or help from others? Could you please describe the application and acceptance procedure?**

[4] Though these forms are in English, you may respond in Dutch of course.
