# Supplementary material for: International Health Electives: defining learning outcomes for a unique experience
Source: BMC Med Educ. 2023 Mar 15;23:157. doi: 10.1186/s12909-023-04124-4 (PMC10015142; doi:10.1186/s12909-023-04124-4)
Supplement: Supplementary file 1 — Additional file 1. [file 12909_2023_4124_MOESM1_ESM.zip › umcg-report.pdf]

2022 - 2023

UMCG Grant Report

Introductory explanation

Dear student,

This report consists of 3 parts:

- Part 1 is for *formal* purposes, in order to be able to transfer the remaining part of the grant (30%). This part is only for *internal use* by the international office and the financial department officer, and not to be shared with any other student.
- Part 2 is intended for *information* purposes, for future students. It is mandatory to fill in this part, but you decide yourself whether we can share the content with students. There is a separate question about this approval in Part *1*.
- Part 3 is in *confidential* and for internal use only, to monitor the quality of our international network and to get better insight on the impact of the international experiences. The information from part 3 will not be shared with your host institution.

Though you might be taking part in group work, or doing the same internship abroad, the international experience is still very individual, so all these forms need to be filled in personally, also in case of joint / group work.

The reporting obligation is part of UMCG grant rules and regulations. (In case of failing to report also the first 70% of the grant can be reclaimed.)

If you have any questions about this report and evaluation procedure, either on the content or the privacy aspects, please turn to fmw.naarhetbuitenland@umcg.nl

UMCG Grant Report

Please fill in and send as Word file. Deadline to hand in this form: 30 days after end date mobility.

Part 1 - formal

**Student**	
First & Family name	
E-mail address	
Student number	

**Departure date and mobility duration**	
Departure date	
Start date mobility	
End date mobility	
Return date	

**Privacy**	
I hereby give permission to the International Office to publish my report on Study Info to inform future students	☐ Yes<br>☐ No
Future students may contact me for additional information[1]	☐ Yes<br>☐ No

**Signature**	
Student name	
Place and date	
Signature[2]	

Required annexes:

- Copy of travel documents, flight tickets, or any other proof to verify start and end date of mobility

[1] If yes, please fill in your e-mail address at the end of Part 2.
[2] Please paste as image/picture.

Part 2 - Content and experience

**1. Study and study item**	
**Study**	☐ Geneeskunde (Medicine)<br>☐ Tandheelkunde<br>☐ Bewegingswetenschappen/Human Movement Sciences/Sports Science)<br>☐ MMIT<br>☐ CPE<br>☐ IMIM<br>☐ ______________________________
**Study item**	☐ Bachelor Thesis Project<br>☐ Semi-arts stage<br>☐ Stage Wetenschap<br>☐ Afstudeerproject (BW/HMS/SS)<br>☐ Individuele Profilering (THK)<br>☐ Research internship (CPE/MMIT/IMIM)<br>☐ ______________________________
**Study item**	
**Number of ECTS**	
**(Sub)discipline[3]**	
**2. Host institution**	
**University/organisation/hospital**	
**Department**	
**City**	
**Country**	
**3. Departure date and mobility duration**	
**Start date mobility**	
**End date mobility**	
**4. Content description and recommendation[4]**	
Programme	
**Please describe your programme, study activities and experiences[5].**	
Advice	
**Would you recommend – unconditionally, conditionally or not at all - future student to do this specific activity at this location? If conditionally, please explain!**	

[3] Only for Geneeskunde, see for list to use https://student.portal.rug.nl/infonet/studenten/umcg/geneeskunde/going-abroad/tarievenmp300816.pdf

[4] Though these forms are in English, you may of course respond in Dutch.

[5] If you have approved to use this part for information purposes to future students, please avoid to mention any names

5. Safety and practical affairs

Travelling

Are there any travelling issues to be worth mentioning? Was there a pick-up service, or did you manage to get to the destination yourself? What about daily transportation from accommodation to workplace?

Accommodation

Did the host institution provide for accommodation, or did you arrange for that yourself? Were you satisfied with the quality of accommodation?

Support, introduction, social

Were you happy with the support at the host institution in general? Was there an introductory programme? Social activities?

Safety

Are there any safety issues to report, both concerning the internship itself, within the host institution, as well as outside, in the daily life, after hours and weekends?

6. Finances

Budget and expenses

For future students: what did your budget estimation look like? Was it realistic? Were there any surprises in reality, positive or negative?

Funding and grants

Any advice for future student in the domain of available grants and additional funding?

7. Any other remarks, comments, advice?

Part 3 – Feedback on quality

(only for internal use and quality assurance purpose, not for publication)

Content evaluation
1 Experience
To what extent did your experience match your expectations[6]?<br><br>1 / 2 / 3 / 4 / 5 / 6 / 7 / 8 / 9 / 10<br>*(Not at all For the full 100%)*
In case of mismatch, please explain!
2 Learning goals
To what extent were you able to achieve your learning goals?<br><br>1 / 2 / 3 / 4 / 5 / 6 / 7 / 8 / 9 / 10<br>*(Not at all For the full 100%)*
In case of a score of 6 or less, please explain!
3 Supervision
Was there enough guidance, supervision and feedback?<br><br>1 / 2 / 3 / 4 / 5 / 6 / 7 / 8 / 9 / 10<br>*(Not at all For the full 100%)*
In case of a score of 6 or less, please explain!
4 Language and communication
Did you engage any language difficulty, in particular in the communication with patients? If so please explain and give advice how to deal with this aspect.

[6] In case you did a 'combicoschap' (Tropical Medicine and Public Health or otherwise) and your experience in the one part was quite different from the other, please give two different marks!

5 Preparation
**To what extent did feel you prepared for this activity?**<br><br>**1 / 2 / 3 / 4 / 5 / 6 / 7 / 8 / 9 / 10**<br>*(Not at all* *For the full 100%)*
**In case of feeling underprepared, please explain!**
6 Added value
**Could you please describe the added value of this specific experience – in this country, at this location - for your (professional) development? E.g. what did you learn (expectedly or unexpectedly) what you probably would not have been able to learn anywhere else?**
**The other way around, could you please elaborate on the added value of your presence and activity for the host institution? What were you able to contribute there?**
**In case of research internships, will the results be published, presented at a conference or otherwise used or distributed?**
**Optional (maybe hard to answer): looking back on this (cultural) experience, do you think it will affect your (professional) plans for the future, choices and/or world view? If yes, in what way?**
7 Special circumstances
**Were there any special circumstances that have affected the course of your internship? If so, please explain!**

8 *In total*
Please give a mark for the overall quality of the internship<br><br>1 / 2 / 3 / 4 / 5 / 6 / 7 / 8 / 9 / 10
Please explain! (In particular in case your mark is 6 or less.)
9 *Any other remarks, comments, concerns or advice?*

Thank you very much!
